# Effects of Paradigm Color and Screen Brightness on Visual Fatigue in Light Environment of Night Based on Eye Tracker and EEG Acquisition Equipment

**DOI:** 10.3390/s22114082

**Published:** 2022-05-27

**Authors:** Peiyuan Tian, Guanghua Xu, Chengcheng Han, Xiaowei Zheng, Kai Zhang, Chenghang Du, Fan Wei, Sicong Zhang

**Affiliations:** 1 School of Mechanical Engineering, Xi’an Jiaotong University, Xi’an 710049, China; tian930724@stu.xjtu.edu.cn (P.T.); hanchengcheng@xjtu.edu.cn (C.H.); hlydx1314@stu.xjtu.edu.cn (X.Z.); zhangkai0912@stu.xjtu.edu.cn (K.Z.); d793660193@stu.xjtu.edu.cn (C.D.); wf3117370016@stu.xjtu.edu.cn (F.W.); zhsicong@xjtu.edu.cn (S.Z.); 2 State Key Laboratory for Manufacturing Systems Engineering, Xi’an Jiaotong University, Xi’an 710049, China

**Keywords:** color paradigm, screen brightness, pupil diameter, θ + α frequency band, visual anti-fatigue index

## Abstract

Nowadays, more people tend to go to bed late and spend their sleep time with various electronic devices. At the same time, the BCI (brain–computer interface) rehabilitation equipment uses a visual display, thus it is necessary to evaluate the problem of visual fatigue to avoid the impact on the training effect. Therefore, it is very important to understand the impact of using electronic devices in a dark environment at night on human visual fatigue. This paper uses Matlab to write different color paradigm stimulations, uses a 4K display with an adjustable screen brightness to jointly design the experiment, uses eye tracker and g.tec Electroencephalogram (EEG) equipment to collect the signal, and then carries out data processing and analysis, finally obtaining the influence of the combination of different colors and different screen brightness on human visual fatigue in a dark environment. In this study, subjects were asked to evaluate their subjective (Likert scale) perception, and objective signals (pupil diameter, θ + α frequency band data) were collected in a dark environment (<3 lx). The Likert scale showed that a low screen brightness in the dark environment could reduce the visual fatigue of the subjects, and participants preferred blue to red. The pupil data revealed that visual perception sensitivity was more vulnerable to stimulation at a medium and high screen brightness, which is easier to deepen visual fatigue. EEG frequency band data concluded that there was no significant difference between paradigm colors and screen brightness on visual fatigue. On this basis, this paper puts forward a new index—the visual anti-fatigue index, which provides a valuable reference for the optimization of the indoor living environment, the improvement of satisfaction with the use of electronic equipment and BCI rehabilitation equipment, and the protection of human eyes.

## 1. Introduction

With the development of science and technology, electronic devices such as computers and mobile phones are more and more integrated with our work and life [1]. Computers are one of the main tools used by government agencies, large enterprises, and small families. On the one hand, these modern technologies, including the Internet and computers, make life so convenient, but on the other hand, they also bring many risks to human health [2]. In daily life, the demand for a large number of computer-related eye tasks is rapidly increasing, and the increase in the amount of these tasks has also led to an increase in visual fatigue [3]. This intensive use of eyes is not only limited to adults, but children are increasingly using computers for learning or entertainment [4]. Not only that, long-term viewing of computer screens can cause visual fatigue and even cause various eye diseases. For this reason, various physical health risks caused by unhealthy eye habits need to be taken seriously by us [5]. At the same time, in recent years, the concept of BCI rehabilitation is also being accepted and explored by more and more people. The relevant literature shows that visual display paradigm stimulation in the BCI is an essential part [6], so it is indispensable to pay attention to relevant visual perception problems. In this, visual fatigue will reduce the training effect and greatly discount it [7]. Therefore, in the actual use of BCI rehabilitation equipment, the subjective and objective combination of visual fatigue and quantitative evaluation to avoid greatly discounting the training and rehabilitation effect of subjects and patients is also a hot research topic.

In terms of eye health, the light environment, the screen brightness of the computer, and the color contrast of the electronic device are in very important positions [8]. In daily life, the light environment is closely related to the physical and mental health of workers [9]. A lot of bright screen brightness will make the eyes quickly produce discomfort [10], meanwhile, a lot of dark screen brightness will reduce the adjustment function of the eyes, will accelerate the generation of asthenopia, and may also produce more profound sequelae [11]. Traditional electronic equipment manufacturers will reduce visual fatigue by reducing the color temperature [12] and adjusting the screen brightness [13]. When the screen brightness is dark, it can indeed reduce the visual fatigue of users [14]. However, some researchers point out that a brighter background can bring better visual effects and is more conducive to the observation of office space users [15,16].

Another important factor of the screen display is brightness contrast [17]. Screen brightness contrast is an index to measure the brightness difference between the observed object and its adjacent background in the display field of vision [18]. Many scholars have found that high brightness contrast often brings a better visual observation experience [15,19], and Tian et al. illustrate that there is an interaction between the brightness and brightness contrast of the screen [20]. At the same time, color is also a very important screen display parameter [21]. The optical primary colors are red, green, and blue. After mixing the three primary colors, the display screen will display the specific color. The three primary colors will be added together to form white which belongs to the colorless system (black, white, and gray) [22]. Some studies have pointed out that different color displays will also bring different visual experiences to users [23]. Relatively few studies thoroughly simulate the night environment (low ambient illumination) to study the visual fatigue caused by watching electronic displays with different color display modes at night. In this study, a subjective questionnaire and objective index measurement were used to study visual fatigue.

Based on the above two variables (screen brightness and screen color display), many BCI visual applications are related to these two variables, such as the stimulation paradigm design in BCI spelling systems and brain-controlled UAV [24,25]. Through this study, the optimal screen brightness and paradigm color can be set, which can improve the recognition efficiency and reduce the fatigue of users. Other related applications are the latest fast brain–computer interface combined with machine vision [26], the mixed reality design of a brain–computer interface based on the industrial environment [27], and the use of an optimized support vector machine classifier under a brain–computer interface to classify the attention load in multi-target tracking tasks [28]. In these relevant applications, the corresponding parameters could be changed to achieve the best user visual experience. Similarly, some researchers have proposed that the EEG and pupil diameter analysis method used in this paper has been used in relevant fields, such as performance evaluation and workload estimation in the training of space remote control robots [29], the use of force feedback equipment to classify objects in the hybrid brain–computer interface [30], in a deep coupling cyclic automatic encoder for alert estimation [31], etc., which shows how combining EEG plus eye movement is an advanced and reliable analysis method.

In our study, low, medium, and dark modes are simulated for the experiment. By adjusting the chroma of the stimulus paradigm, the brightness contrast of the four-color patterns is consistent in the same background mode (red on white, green on white, blue on white, and black on white). In the same brightness mode and the same brightness contrast, the degree of visual fatigue is affected by different color modes. To study the influence of screen color display parameters–screen brightness parameters on visual fatigue, this study adjusted the chromaticity of the color parameters in three screen brightness mode conditions (to achieve the same brightness contrast of four groups of colors in the same screen brightness mode, bright mode: 0.383, medium mode: 0.500, dark mode: 0.400). In addition, during this study, the screen brightness was controlled in 422.6 CD/m^2^ (bright mode), 287.6 CD/m^2^ (medium mode), and 52.4 CD/m^2^ (dark mode). Brightness contrast is defined as the ratio of the brightness of the color stimulus paradigm to the brightness of the white background (Michelson contrast). Environmental lighting is another important factor affecting visual performance. To study the influence of different color groups on the subjects under different screen brightness at night, the environmental illumination is controlled below 3 lx. This study aims to solve the following problems: how color display parameters affect visual fatigue, how the color screen brightness affects visual fatigue, and the interaction between the color display parameters and screen brightness when using electronic devices in a low ambient brightness at night. In the rest of the paper, the second part introduces the details of the experiment, the third part explains the experimental data analysis, the fourth part discusses the data results, and the fifth part is the conclusion and application.

1. Aiming at the increasingly serious problem of visual fatigue caused by the use of electronic equipment at night, this experiment combined subjective (Likert scale) and objective (EEG + pupil data) methods to explore the impact of the combination of different colors and screen brightness on human visual fatigue in the dark environment.
2. Most articles in the same field are analyzed and studied based on subjective questionnaires, and a few are combined with EEG data or eye movement data. In this paper, EEG and eye movement are combined to further improve the proportion of objective data, make the results more convincing, and make visual fatigue more objectively quantified.
3. This paper creatively puts forward a new index to measure visual fatigue. This index combines subjective and objective indexes, and then evaluates their fatigue impact on human eyes by scoring the corresponding color-brightness modes. It also has a good reference value for the use of electronic equipment at night and the design of electronic equipment in factories.

## 2. Materials and Methods

### 2.1. Subjects

**Figure 1:**
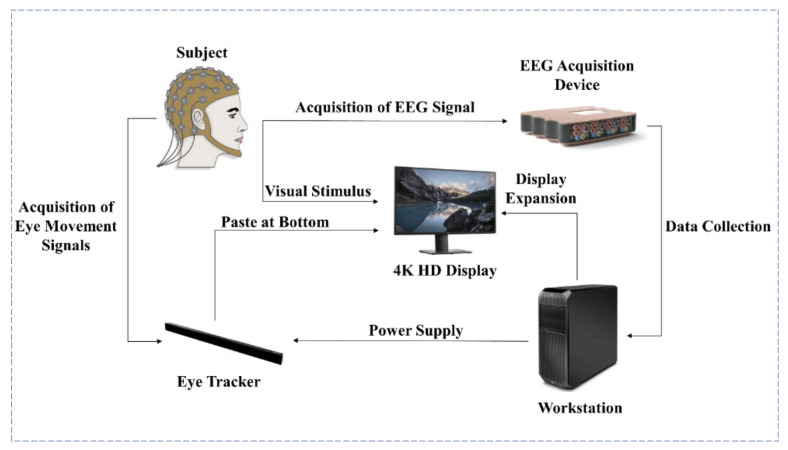
Experimental system.

**Figure 2:**
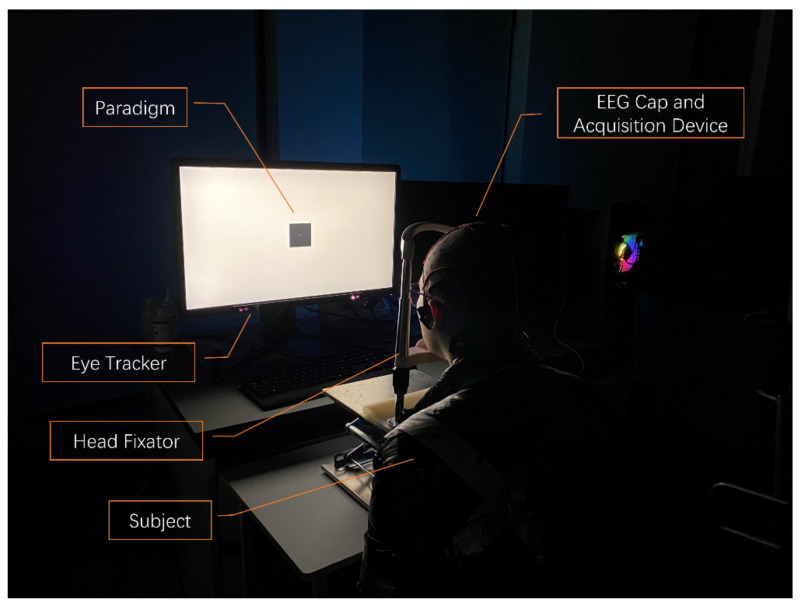
Experimental scene.

The design of the whole experiment and the real experimental scene is shown in Figure 1 and Figure 2, respectively. Fifteen subjects (ten males and five females) were recruited for this experiment. All subjects were aged between 21 and 27 years old (mean = 24.67 years old, std = 1.40 years old), and their visual acuity or corrected visual acuity was above 1.0 (mean = 1.15, std = 0.11). The subjects could not wear contact lenses and had no eye diseases, such as abnormal refraction, dry eye, color blindness, and strabismus. The subjects were informed to sleep for at least 7 h within 24 h before the experiment. The use time of electronic products was only 8 h, as far as possible, and participants did not eat irritant food and drinks. In the actual experiment, the following methods were used for helping experiment’s execution: 1. Reminded the subjects of relevant precautions 12 h before the beginning of the experiment. 2. Before the beginning of the experiment, asked the subjects about their state, and observed whether they have conditions that were not suitable for the experiment which include, but were not limited to, being drunk, yawning, and so on. If the state of the subject was not suitable, we rearranged another time to do the experiment. All subjects were given informed consent following the Helsinki Declaration and approved by the institutional review committee of Xi’an Jiaotong University.

### 2.2. Signal Acquisition

The g.tec (g.tec, Schiedlberg, Austria) EEG (Electroencephalogram) equipment and eye tracker are utilized to collect EEG and pupil signals to detect visual fatigue, and the Likert scale is provided to collect visual fatigue scale and subjective preference score. EEG signal has been used in many aspects, such as dynamic optimization of spelling system [32], or four-dimensional spelling device with multiple signal coupling [33], to help paralyzed patients realize brain-controlled typing, in which brain fatigue is also one of the reference cores of the design. Electrooculogram is also used to detect various types of asthenopia and the same Likert scale has been widely used to measure the subjective indicators of physiology and psychology. As this experiment is aimed at the visual area, according to the 10–20 system, the electrodes are arranged in the occipital region of the brain, which are PO3, PO4, POz, O1, O2, and Oz, respectively, to record the corresponding EEG signals. The FPz of the forehead is the grounding electrode, and the A1 of the left earlobe is the reference electrode [34]. The signal acquisition consists of two parts, one is the EEG acquisition system (g.tec, Schiedlberg, Austria), for which the sampling rate is 1200 Hz, which consists of g.USBamp acquisition and processing system and g.GAMMAbox active electrode system; the other is Tobii Pro-fusion (Tobii, Stockholm, Sweden), for which the sampling rate is 120 Hz, which records eye movement signals. Furthermore, an online band-pass filter with a bandwidth of 2–100 Hz is utilized to eliminate artifacts, and an off-line notch filter with a bandwidth of 48–52 Hz is applied to eliminate power line interference.

### 2.3. Stimulus Designs

In this experiment, a 4K high-definition display (ROG XG27UQ, ASUS, Taipei, China) with a resolution of 3840 × 2160 and a refresh rate of 60 Hz was equipped. The illumination of the screen and the chromaticity value of the paradigm and its background were measured by the HOPOO spectral brightness analyzer (OHSP-350L, Hangzhou HOPOO Optical Color Technology Co., Ltd., Hangzhou, China). In the experiment, the subjects were asked to sit 55 cm away from the center of the display and the eyes of participants were flat with the center of the display. The angle of view of paradigm stimulation was 4°, and the diameter was 148 pixels, which was in line with the design ideas of relevant literature [35,36]. Meanwhile, the eye tracker was attached to the bottom part of the monitor, almost hidden, to minimize the interference with the test object. During the whole experiment, although the screen brightness and paradigm color changed, the luminance contrast of all paradigm stimuli and background was artificially controlled to be constant to control variables. The stimulus paradigms were adjusted by MATLAB (MathWorks, Natick, MA, United States) with the Psychophysics Toolbox [37].

**Figure 3:**
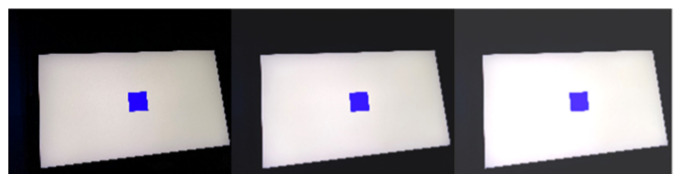
The contrast of three kinds of brightness in the same color paradigm.

As shown in Table 1, there are three groups of four paradigms in this experiment. According to the screen brightness, the three groups are divided into 0% brightness, 50% brightness, and 100% brightness. There are four color paradigms of red, green, blue, and black in each group with the white background, and the color order in each group is fixed. During the experiment, the order of the three groups is randomly selected by the subjects. Figure 3 shows the contrast of the same paradigm color under different brightness contrast modes.

### 2.4. Experimental Procedure

In order to avoid the influence of circadian rhythm, the experiment was set starting at 8:00 p.m. every day, however, because everyone had different rest times between each round of experiments, the end time was slightly different. To conclude, the experimental time of each of the 15 subjects was less than 2.5 h after statistics which means the experiments were controlled between 8:00 p.m. and 10:30 p.m. every day to avoid the possible impact of circadian rhythm on the experiment. Each subject was tested 12 times (three groups), each experiment corresponding to a paradigm, the order of the three groups were selected by the subjects. Before each round of the experiment, the subjects were asked about the degree of subjective visual fatigue, and the Likert scale was used to record. In the experiment, the pupil diameter data of the subjects were recorded by an eye tracker (Tobii, Stockholm, Sweden) with a sampling rate of 120 Hz. The break times between each two different paradigms is about 9 min, there are 12 paradigms (mean = 98.86 min, std = 4.20 min) in total. Each experiment included 23 trials that each lasted 5 s, with an interval of 0.5 s between two trials, in which the first three were pre-experimental trials. A head fixator was used to ensure the stability of the subjects’ heads and the accuracy of signal acquisition. The subjects were staring at the white static “x” mark on the black background. At this time, there was no interference between the pupil light reflection and the ambient light [38]. The EEG data and eye movement data here can be used as the normalized reference values for subsequent trials. The next 20 trials were formal trials. The color scintillation paradigm was run, and a white “x” mark appeared in the center of the paradigm to help fix the line of sight [35]. At the same time, the data were recorded by various devices. At the end of the trials, the subjects were asked about the degree of subjective visual fatigue and scored the degree of subjective preference, which were also recorded with the Likert scale. At this point, one of the 12 rounds of the experiment was completed, and the subjects had a good rest until the next round of the experiment, which means the rest time was controlled by the subjects themselves. The whole experimental time of each subject was about 120 min, depending on the rest time.

### 2.5. Experimental Index

#### 2.5.1. Visual Fatigue Scale (VFS) and Subjective Preference (SP)

The Likert scale is a common summation rating scale in which items are scored by summation. It is meaningless to only look at individual item scores. The scale consists of a set of statements. Each sentence has five answers: “very disagree”, “disagree”, “not necessarily”, “agree”, and “very agree”, which are 1, 2, 3, 4, and 5 points, respectively. This experiment is more specific, and the score given by the subjects is 1 to 10 points from very disagree to very agree. The final score of each person consulted is the sum of the scores of all questions. The total score can indicate the strength of subjective attitudes and different feelings on the scale.

According to the questionnaire developed by Xie [39], this experiment made some modifications to make it more in line with the experimental environment. There are seven questions: 1. It is hard for me to see the screen clearly. 2. I have a strange feeling in my eyes. 3. I have sore eyes, such as acerbity, tingling, swelling, etc. 4. The brightness of the screen numbs my eyes. 5. Looking at the screen, I feel dizzy and fuzzy. 6. I feel a headache. 7. Overall preference after the experiment. The first six questions are the scores of visual fatigue scale (VFS) indicators, and the scores of each question are increased from 1 (very disagree) to 10 (very agree). The final VFS score is the total score. The answer to the seventh question is: 1 (especially dislike), 2 (dislike), 3 (average), 4 (like), and 5 (especially like). This score is the final score of the subjective preference (SP) index.

#### 2.5.2. Pupil Diameter (PD)

Pupil size varies with age, race, refractive status, light intensity, distance of target, and emotion. It is generally 2–5 mm, with an average of about 4 mm. The limit of pupil narrowing or dilation was 1.5 mm and 8 mm, and the difference between the two eyes was less than 0.25 mm. Pupil size can be used to measure eye fatigue, which has a strong correlation with the severity of the disease [40]. By recording the pupil size of the subjects, this experiment objectively quantifies the eye fatigue of the subjects from the perspective of visual perception. All pupil data will be normalized in the subsequent parts.

As mentioned above, each round of the experiment is composed of 23 trials. With the increase of experimental time, the visual fatigue may be aggravated. Therefore, in addition to the pre-experimental trials, the remaining trials in each round are divided into four levels, level 1 (4–8 trials), level 2 (9–13 trials), level 3 (14–18 trials), and level 4 (19–23 trials) [41]. Level 1 represents the least fatigued state at the beginning, and level 4 denotes the most fatigued state at the end [41,42].

#### 2.5.3. θ + α Frequency Band Data

The processing method of EEG data is to use canonical correlation analysis (CCA), CCA is a nonparametric multivariable method to reveal the underlying correlation between two sets of multidimensional variables. In this study, EEG X is the six-electrode channel signal in each trial, which is selected as a set of variables to calculate the canonical correlation coefficient with the generated reference signals $Y_{i}$, in which $Y_{i}$ denotes the composition of sine and cosine pairs that are constructed at the reference frequency $f_{i}$ (i = 1, 2,…, N):

(1) $$Yi=\left(\begin{array}{c} sin\left(2\pi f_{i}t\right)\\ cos\left(2\pi f_{i}t\right) \end{array}\right),\;t=\frac{1}{F_{S}}\;,\ldots ,\;\frac{N}{F_{S}}\;$$

(2) $$\rho_{i}=\underset{w_{x},w_{y}}{\max}\frac{E\left[w_{x}^{T}XY_{i}^{T}w_{yi}\right]}{\sqrt{E\left[w_{x}^{T}XX^{T}w_{x}\right]E\left[w_{yi}^{T}Y_{i}Y_{i}^{T}w_{yi}\right]}}\;$$

Using two sets of signals X and $Y_{i}$, the goal is to find two linear projection vectors $w_{x}$ and $w_{yi}$ so that the two groups of linear combination signals $w_{x}^{T}X$ and $w_{yi}^{T}Y_{i}$ have the largest correlation coefficients. Additionally, the maximum correlation coefficient $\rho_{i}$ between X and $Y_{i}$ can be considered as the response to the stimulation paradigm at the reference frequency $f_{i}$ in visual evoked potentials. Compared to the traditional EEG signal processing method similar to Fourier Transform, the Array signal processing method CCA uses channel covariance information, which may improve the signal-to-noise ratio [43,44]. In addition, CCA is widely used due to its high efficiency, robustness, and simple implementation [45,46]. A band-pass filter of 3–45 Hz was carried out to remove low-frequency drift and high-frequency interference. After filtering and certain data screening, the CCA amplitude corresponding to the 4–7 Hz data of the θ frequency band was extracted and accumulated. Similarly, the required data of 8–13 Hz corresponding to the α frequency band was also extracted after processing, and all the above data were accumulated [47]. This is the EEG data for this experiment. Similarly, it will be normalized later and the trials are still classified as four levels.

### 2.6. Statistical Analysis

SPSS 22.0 software (IBM, Armonk, NY, USA) was used for statistical analysis, and one-way or two-way repeated-measures analysis of variance (ANOVA) with significance < 0.05 was used to analyze the above four indicators (VFS, SP, pupil diameter, θ + α Band) of 12 paradigms at level 1 and level 4. In this experiment, each subject takes one round trial, and there are 12 different groups of stimuli in each round. There are two independent variables: screen brightness and stimulus paradigm color. The screen brightness is divided into three kinds (0%, 50%, 100%), and the stimulus paradigm color is divided into four kinds (blue, black, green, and red). There are four dependent variables: subjects’ subjective fatigue score (VFS), subjects’ subjective preference score (SP), EEG θ + α Band response, and pupil diameter (SP). Shapiro–Wilk normal distribution test was finished before data processing. In the experiment, it was inevitable that sometimes there was the impact of excessive blinking on pupil diameter and head shaking on EEG data acquisition. In the SW test, there were a few singular points which occasionally appeared. After the KS test, the data that did not meet the conditions (the data less than 0.05 in the SW test) was discarded, and then the corresponding data analysis was carried out. All the data of post-hoc tests are listed in Appendix A.

## 3. Results

### 3.1. Pre-Experimental Trials

**Figure 4:**
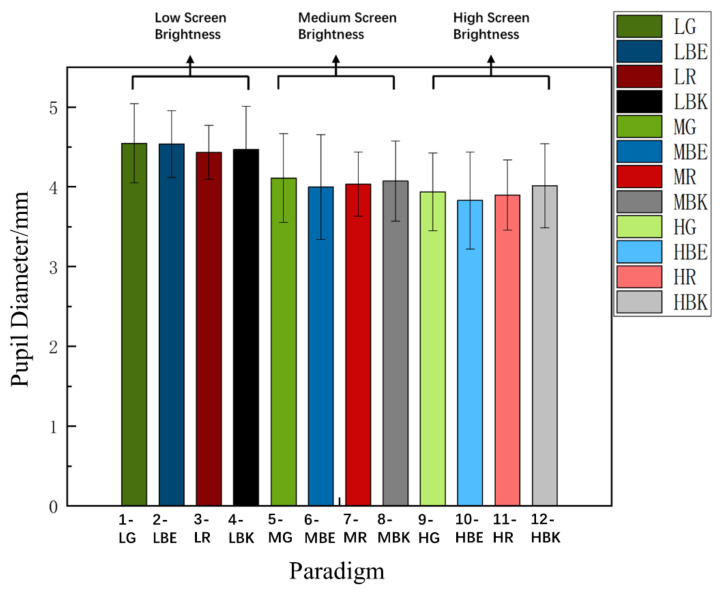
Comparison of the mean values and SD of pupil diameter for 12 paradigms over 15 subjects. Statistics were assessed by one-way repeated-measures ANOVA.

**Figure 5:**
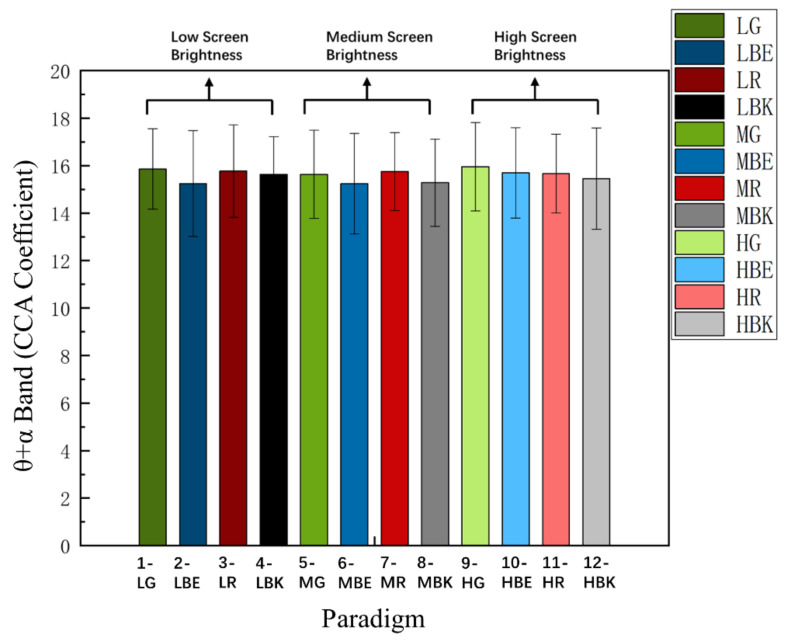
Comparison of the mean values and SD of θ + α band for 12 paradigms over 15 subjects. Statistics were assessed by one-way repeated-measures ANOVA.

In the experiment, we assume that the mental fatigue of each subject was the same at the beginning of each experiment. As there was enough rest time between every two experiments, the 12 paradigms were divided into three groups according to the screen brightness. The experimental order of the three groups of brightness modes was randomly selected by the subjects. Therefore, in order to test this hypothesis, we estimated the initial mental fatigue values of 12 experiments corresponding to 12 paradigms, in which the initial fatigue value was to prepare for the normalization of the subsequent objective data. The average fatigue level of the first three trials was considered to be the initial mental fatigue of this experiment because the test of the first three stimulations was to watch a white “x” cursor in the black background without flashing. Figure 4 and Figure 5 show the related comparison of the pupil diameter and θ + α band for twelve paradigms over fifteen subjects. One-way repeated-measures ANOVA was used to test the mean diameter of the pupil and the average amplitude difference of the θ + α band in the first three trials. As shown in Figure 4, there is a certain difference in pupil diameter, and the difference is not significant in the θ + α band [Greenhouse–Geisser correction: F_(3.526, 49.367)_ = 11.989, *p* < 0.001 *** for the pupil diameter; F_(5.326, 74.558)_ = 1.285, *p* = 0.278 for the θ + α band]. At the same time, post-hoc tests were also made to prove that there is a significant difference in pupil diameter under the same color but different brightness (the pupil diameter is the largest at the low brightness mode), which is also supported by the data in Figure 4. The results of the frequency band analysis verify our hypothesis. As for the pupil diameter, we conducted a more in-depth analysis. According to the different screen brightness, we divided the twelve paradigms into three groups by different screen brightness modes. Each group has four colors (green, blue, red, and black) and conducted one-way repeated measures ANOVA for each group again [Greenhouse–Geisser correction: F_(2.536, 35.498)_ = 1.631, *p* = 0.205 for the low brightness mode; F_(1.840, 25.765)_ = 0.529, *p* = 0.581 for the medium brightness mode; F_(1.422, 19.915)_ = 0.662, *p* = 0.477 for the high brightness mode]. Therefore, we summarize two points: first, at the same brightness, the mental fatigue of the subjects at the beginning of each experiment is the same and can be trusted; second, pupil diameter is sensitive to the brightness of the screen, but the EEG of θ + α band is not sensitive to screen brightness, as Figure 5 shows.

### 3.2. Comparison of Likert Scale

**Figure 6:**
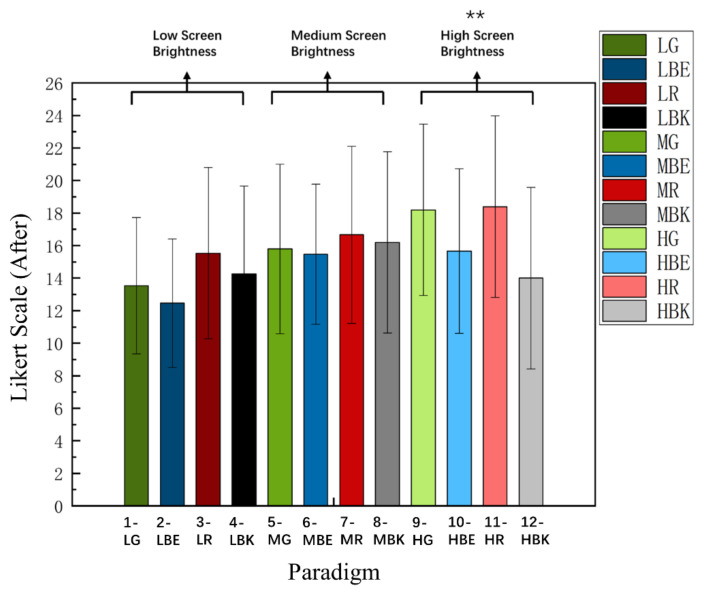
Comparison of the mean values and SD of Likert Scale (VFS) for 12 paradigms over 15 subjects after each run of the experiment. Statistics were assessed by one-way repeated-measures ANOVA. ** *p* < 0.01.

Figure 6 shows the subjective visual fatigue scores of 15 subjects after the experiment. ANOVA analysis showed that there was a significant difference between the score of subjective visual fatigue and different stimulus paradigms after the experiment [F_(5.582, 78.145)_ = 3.044, *p* = 0.012 *]. Meanwhile, two-way repeated-measures ANOVA showed that the interaction of two factors of “screen brightness” and “paradigm color” yielded significance in the visual fatigue scale [F_(4.515, 63.217)_ =2.649, *p* = 0.035 *]. For the in-depth analysis, 12 stimulus paradigms were divided into three groups by the same brightness and different colors (low brightness (paradigm 1, 2, 3, 4), medium brightness (paradigm 5, 6, 7, 8), high brightness (paradigm 9, 10, 11, 12)). The low brightness group [F_(2.529, 35.405)_ = 1.855, *p* = 0.163] and medium brightness group [F_(2.291, 32.073)_ = 0.300, *p* = 0.772] had no significant difference, but the high brightness group [F_(2.421, 33.897)_ = 6.875, *p* = 0.002 **] and further analysis showed that there were significant differences (Bonferroni Post-Hoc Analysis, *p* = 0.003 **) between paradigm 10 (VFS = 14) and paradigm 11 (VFS = 18.4), (Bonferroni Post-Hoc Analysis, *p* = 0.044 *) and between paradigm 10 (VFS = 14) and paradigm 12 (VFS = 18.2), indicating that the subjects preferred paradigm 10 (HBK) to paradigm 11 (HR) or paradigm 12 (HG) in the high brightness mode.

Meanwhile, the 12 stimulus paradigms were divided into four groups (green group (paradigm 1, 7, 12), blue group (paradigm 2, 6, 9), red group (paradigm 3, 8, 11), and black group (paradigm 4, 5, 10)) by the same color and different brightness. The green group [F_(1.589, 22.243)_ = 6.918, *p* = 0.007 **] showed significant differences. Further analysis revealed that there were differences (Bonferroni Post-Hoc Analysis, *p* = 0.007 **) between paradigm 1 and paradigm 12; the higher the score was, the more the participants felt visual fatigue under the combination of current brightness and current color. The average score of paradigm 1 was 13.5, and that of paradigm 12 was 18.2. The participants preferred paradigm 1 (LG).

More specific analysis of the other three color-groups were shown in the following: blue group [F_(1.709, 23.922)_ = 2.834, *p* = 0.086]; red group [F_(1.732, 24.246)_ = 2.225, *p* = 0.135]; black group [F_(1.855, 25.965)_ = 0.921, *p* = 0.404], which means there was no significant difference between them and no need for further analysis.

**Figure 7:**
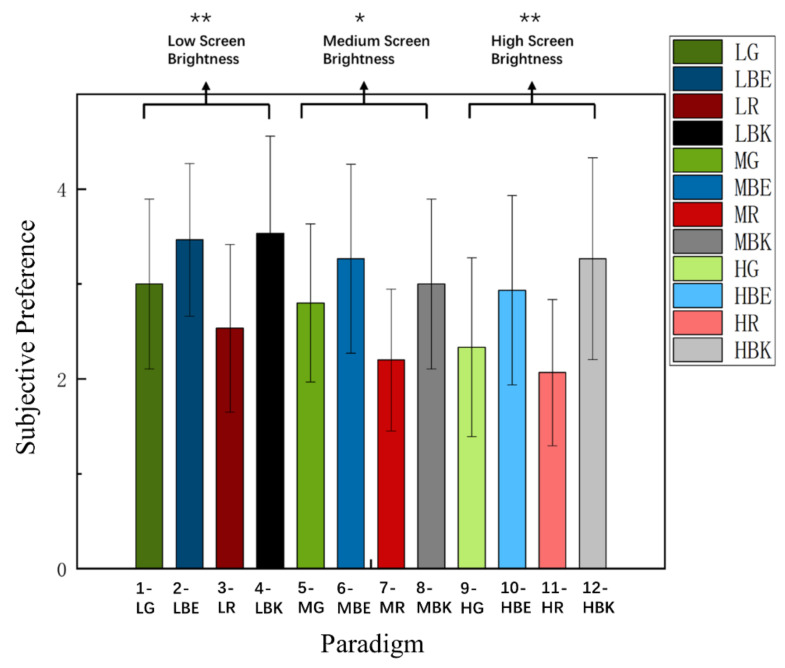
Comparison of the mean values and SD of SP for 12 paradigms over 15 subjects. Statistics were assessed by one-way repeated-measures ANOVA. ** *p* < 0.01; * *p* < 0.05.

As shown in Figure 7, it can be found that there are differences in SP scores among different paradigms [F_(5.433, 76.059)_ =5.284, *p* < 0.001 ***]. Two-way repeated-measures ANOVA revealed that the interaction of the two factors of “screen brightness” and “paradigm color” yielded significance in subjective preferences [F_(3.893,54.499)_ = 4.383, *p* = 0.004 **]. The analysis result of the low brightness group [F_(2.009, 28.130)_ = 5.734, *p* = 0.008 **] showed significant differences that led the further analysis. There were significant differences (Bonferroni Post-Hoc Analysis, *p* = 0.046 *) between paradigm 2 (SP = 3.5) and paradigm 3 (SP = 2.5), which indicated that the subjects preferred paradigm 2 (LBE) to paradigm 3 (LR) in the low brightness mode.

The same as before, there was a significant difference in the medium brightness group [F_(2.683, 37.561)_ = 4.466, *p* = 0.011 *], to be more specific, there were significant differences (Bonferroni Post-Hoc Analysis, *p* = 0.004 **) between paradigm 6 (SP = 3.3) and paradigm 8 (SP = 2.2), which indicated that the subjects preferred paradigm 6 (MBE) to paradigm 8 (MR) in the medium brightness mode.

Similarly, in the high brightness group [F_(2.176, 30.459)_ = 7.499, *p* = 0.002 **], there was a significant difference. Further analysis showed that there were significant differences (Bonferroni Post-Hoc Analysis, *p* = 0.003 **) between paradigm 9 (SP = 2.9) and paradigm 11 (SP = 2.1), as well as (Bonferroni Post-Hoc Analysis, *p* = 0.001 **) between paradigm 10 (SP = 3.3) and paradigm 11 (SP = 2.1), indicating that the subjects preferred paradigm 9 (HBE) and paradigm 10 (HBK) to paradigm 11 (HR) in the high brightness mode.

Meanwhile, the 12 stimulus paradigms were divided into four groups (green group (paradigm 1, 7, 12), blue group (paradigm 2, 6, 9), red group (paradigm 3, 8, 11), and black group (paradigm 4, 5, 10)) by the same color and different brightness. The green group [F_(1.662, 14.962)_ = 4.442, *p* = 0.036 *] showed significant differences. Further analysis revealed that there were differences (Bonferroni Post-Hoc Analysis, *p* = 0.045 *) between paradigm 1 and paradigm 12, the higher the score was, the more the participants liked the combination of current brightness and current color. The average score of paradigm 1 was 3, and that of paradigm 12 was 2. The participants preferred paradigm 1 (LG).

Furthermore, the analysis data showed few significances of three other color-groups separately: blue group [F_(1.603, 14.429)_ = 1.000, *p* = 0.375]; red group [F_(1.854, 16.686)_ = 0.265, *p* = 0.754], and black group [F_(1.597, 14.377)_ = 2.629, *p* = 0.114], in which there was no in-depth analysis because of there being no significant difference.

### 3.3. Comparison of Pupil Diameter Index

**Figure 8:**
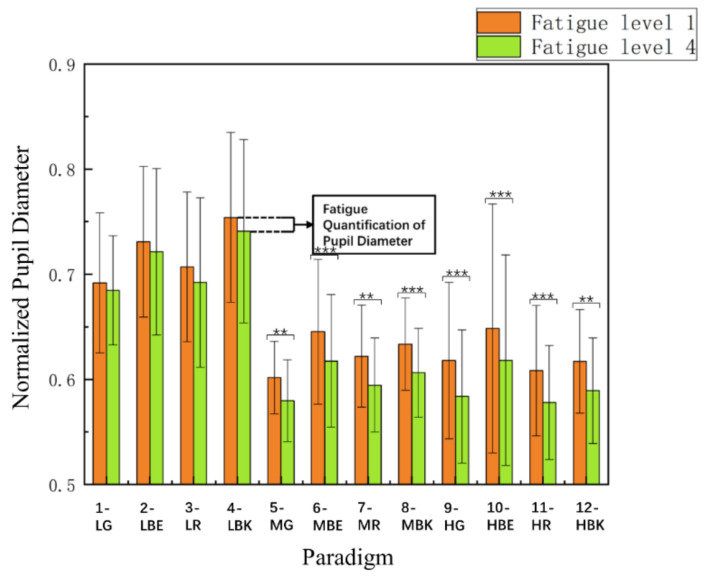
Comparison of the mean values and SD of normalized pupil diameter index between fatigue level 1 and fatigue level 4 for 12 stimulus paradigms over 15 subjects. Statistics were assessed by one-way repeated-measures ANOVA. *** *p* < 0.001; ** *p* < 0.01.

For the convenience and standardization of data analysis, in each experiment, the first three experiments (a total of 23 paradigm stimulations, the first three trials were prepared for standard normalization, and the last 20 trials were formal experimental stimuli) were designed to normalize the pupil diameter data of experimental participants. Figure 8 shows the normalized pupil diameter index between fatigue level 1 and fatigue level 4 of 12 stimulus paradigms among 15 subjects. Two-way repeated-measures ANOVA revealed that the interaction of the two factors of “stimulus paradigm” and “fatigue level” had no significance in the normalized pupil diameter index [F_(2.375, 33.248)_ = 1.813, *p* = 0.173]. Meanwhile, two-way repeated-measures ANOVA illustrated that the interaction of the two factors of “screen brightness” and “paradigm color” yielded significance in the pupil diameter among level 1 and level 4, respectively [F _(3.893,54.499)_ = 4.383, *p* = 0.004 **] and [F_(2.271,31.790)_ = 9.549, *p* < 0.001 ***]. Subsequently, one-way repeated measures ANOVA pointed out a significant difference in the pupil diameter index among twelve paradigms at fatigue level 1 [F_(2.576,36.058)_ = 8.022, *p* < 0.001 ***] and fatigue level 4 [F_(2.936, 41.099)_ = 14.934, *p* < 0.001 ***], respectively. From the above analysis, it can be seen that there is no significant difference in the pupil diameter index under the “stimulation paradigm” and “fatigue level”, but it can be seen from Figure 8 that the pupil diameter index under a low background brightness is the largest, regardless of whether it is in fatigue level 1 or fatigue level 4; on the contrary, there is no significant difference between the medium background brightness and the high background brightness. In conclusion, visual fatigue was the lightest in the low brightness mode during the whole experiment.

In the in-depth analysis, 12 stimulus paradigms were divided into three groups by the same brightness and different colors as before (low brightness (paradigm 1, 2, 3, 4), medium brightness (paradigm 5, 6, 7, 8), high brightness (paradigm 9, 10, 11, 12)). The low brightness group [F_(1.355, 18.974)_ = 0.148, *p* = 0.780] showed no significant differences. Similarly, there was no significant difference in the medium brightness group [F_(1.163, 16.276)_ = 0.097, *p* = 0.797] and high brightness group [F_(1.122, 15.714)_ = 0.857, *p* = 0.381]. Therefore, further analysis was not necessary.

Meanwhile, the twelve stimulus paradigms were divided into four groups (green group (paradigm 1, 7, 12), blue group (paradigm 2, 6, 9), red group (paradigm 3, 8, 11), and black group (paradigm 4, 5, 10)) by the same color and different brightness. The green group [F_(1.614, 22.593)_ = 16.674, *p* < 0.001 ***] showed significant differences. Further analysis revealed that under the same color stimulus paradigm, there were significant differences in the pupil diameter data after different brightness experiments. There were significant differences (Bonferroni Post-Hoc Analysis, *p* < 0.001 ***; Bonferroni Post-Hoc Analysis, *p* < 0.001 ***) between paradigms 1 and 12, and paradigms 7 and 12, respectively. It can be concluded that the experimental participants were more prone to visual fatigue in a high brightness mode when they were stimulated by the green paradigm under different screen brightness.

Meanwhile, the blue group [F_(1.358, 19.005)_ = 4.469, *p* = 0.038 *] inferred that there was a significance. There were significant differences (Bonferroni Post-Hoc Analysis, *p* = 0.004 **; Bonferroni Post-Hoc Analysis, *p* = 0.001 **) between paradigms 2 and 9, and paradigms 6 and 9, respectively. It can be induced that the experimental participants were more prone to visual fatigue in the high brightness mode when they were stimulated by the blue paradigm with different background brightness.

Similarly, the red group [F_(1.402, 19.634)_ = 21.919, *p* < 0.001 ***] explained significant differences. There were significant differences (Bonferroni Post-Hoc Analysis, *p* < 0.001 ***; Bonferroni Post-Hoc Analysis, *p* < 0.001 ***) between paradigms 3 and 11, and paradigms 8 and 11, respectively. It can be summed up that the experimental participants were more prone to visual fatigue in the high brightness mode when they were stimulated by the red paradigm with different background brightness.

Finally, the black group [F_(1.480, 20.715)_ = 34.599, *p* < 0.001 ***] illustrated that there was a significance. There were significant differences (Bonferroni Post-Hoc Analysis, *p* < 0.001 ***; Bonferroni Post-Hoc Analysis, *p* < 0.001 ***) between paradigms 4 and 10, and paradigms 5 and 10, respectively. It could also be concluded that the experimental participants were more prone to visual fatigue in the high brightness mode when they were stimulated by the black paradigm with different background brightness.

To summarize, under the same brightness mode, different color paradigm stimulation does not cause a significant difference in the pupil diameter index, that is to say, the degree of the visual fatigue of the subjects is unrelated to the color of the stimulation paradigm. On the contrary, under the same color paradigm stimulation, the pupil diameter index is directly affected by the background brightness mode, not only in the low brightness background, the eyes of subjects are more relaxed, and during the experiment, the subjects are more likely to feel visual fatigue in the high brightness mode.

### 3.4. Comparison of θ + α Index

**Figure 9:**
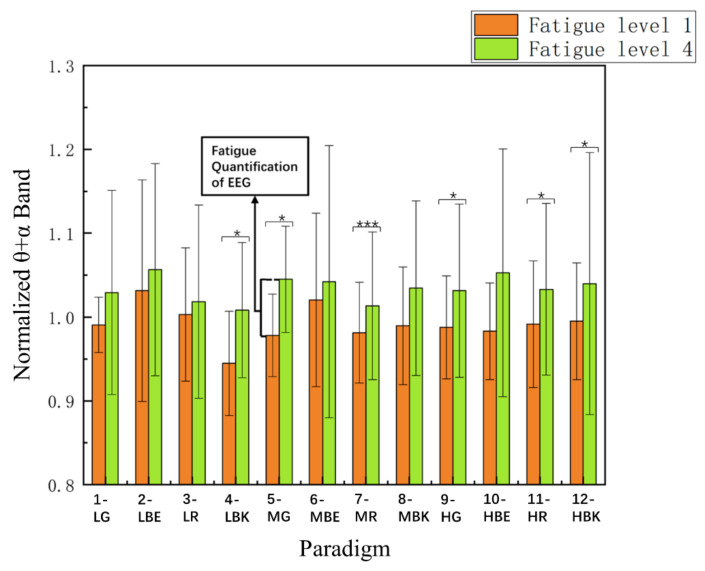
Comparison of the mean values and SD of normalized θ + α index between fatigue level 1 and fatigue level 4 for 12 stimulus paradigms over 15 subjects. Statistics were assessed by one-way repeated-measures ANOVA. *** *p* < 0.001; * *p* < 0.05.

The θ + α frequency band index is an objective quantitative index of the EEG signal for the visual fatigue of subjects [41,42]. According to the baseline data of the first three trials of each paradigm according to each subject, the standardized θ + α frequency band index of each paradigm is calculated. Figure 9 shows the normalized θ + α frequency band index between fatigue level 1 and fatigue level 4 of 12 paradigm stimulation modes among 15 subjects.

Two-way repeated-measures ANOVA indicated that the interaction of the two factors of the “stimulus paradigm” and “fatigue level” was non-significant in the normalized θ + α index [F_(4.388, 61.433)_ = 1.170, *p* = 0.334]. The factor of the “stimulus paradigm” had an insignificant effect on the θ + α index [Greenhouse–Geisser F_(3.449, 48.288)_ = 2.658, *p* = 0.051], and the factor of the “fatigue level” had an insignificant effect on the θ + α index [F_(1.000, 14.000)_ = 0.006, *p* = 0.938]. Meanwhile, two-way repeated-measures ANOVA illustrated that the interaction of the two factors of the “screen brightness” and “paradigm color” yielded significance in the θ + α index among level 1 and level 4, respectively [F_(3.258,45.615)_ = 1.631, *p* = 0.192] and [F_(2.763,38.678)_ = 0.849, *p* = 0.467].

It could be clearly seen that the changing trend of the 12 paradigms is the same, they all increase from fatigue level 1 to fatigue level 4, and the change variables (Mean = 0.042 ± SD = 0.017) tend to be the same. It also can be seen that the objective index of the θ + α frequency band EEG signal induced by the 12 paradigms is unrelated to the screen brightness and paradigm color. All of them can cause almost the same amount of visual fatigue.

### 3.5. Visual Anti-Fatigue Index

In order to unify the index and measure visual fatigue more intuitively, a new definition named the visual anti-fatigue index (VAI) is proposed, which combines the four indexes mentioned earlier in this paper. Considering the previous literature [48,49] and the practical experience in the experiment, the relevant parameters corresponding to the four indexes are determined:

(3) $$A=\sum_{i=1}^{n}a\times \left(PD_{level1}{}_{i}-PD_{level4}{}_{i}\right)$$

(4) $$B=\sum_{i=1}^{n}b\times (Band_{level4}{}_{i}-Band_{level1}{}_{i})$$

(5) $$C=\sum_{i=1}^{n}c\times \left(VFS_{after}{}_{i}-VFS_{before}{}_{i}\right)\;$$

(6) $$D=\sum_{i=1}^{n}d\times \left(-1\right)\times SP_{i}\;$$

(7) $$VAI=\frac{A+B+C+D}{n}\;$$

where i represents the i-th subject, n denotes the number of subjects in total, *a* = 0.5, *b* = 0.3, *c* = 0.15, *d* = 0.05, $PD_{level1}$ is the normalized pupil diameter at fatigue level 1, $PD_{level4}$ is the normalized pupil diameter at fatigue level 4, $Band_{level1}$ is the normalized α + θ frequency band amplitude value of fatigue level 1, $Band_{level4}$ is the normalized α + θ frequency band amplitude value of fatigue level 4, $VFS_{before}$ is the subjective visual fatigue score of subjects on Likert scale before the experiment, $VFS_{after}$ is the subjective visual fatigue score of subjects on the Likert scale after the experiment, and SP is the subjective preference score.

The weight coefficients a (pupil diameter weight), *b* (θ + α band CCA coefficient weight), *c* (visual fatigue scale weight), and *d* (subjective preference weight) were set as 0.5, 0.3, 0.15, and 0.05, subjectively. The following are reasons for this setting: firstly, subjective factors are more easily affected by the mood, state, sleep, and other uncertain factors of subjects; therefore, the subjective weight should be less than the objective weight. Secondly, the questionnaire of VFS refers to other scientific works, which is more in line with general cognition and makes VFS more credible than SP. Thirdly, it is obvious that one of the most intuitive manifestations of visual fatigue is a change in the pupil diameter, thus PD deserves the maximum weight. Fourthly, the weight of the θ + α band is set between the weight of PD and the weight of VFS.

**Figure 10:**
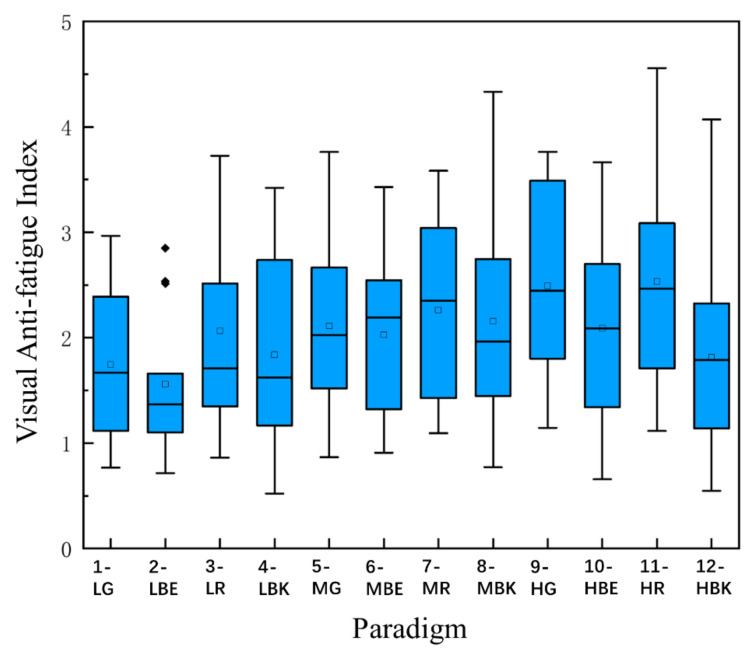
Comparison of the mean values and SD of the visual anti-fatigue index for 12 stimulus paradigms over 15 subjects. Hollow square points represent average values, solid points represent outliers.

The lower the visual anti-fatigue index, the better the anti-fatigue effect of this mode. It can be seen from Figure 10 that the general anti-fatigue performance of the low screen brightness mode is better than that of the medium and high screen brightness modes [F_(5.625, 78.744)_ = 3.347, *p* = 0.006 **]. Further analysis under the same brightness but different colors situation, which includes the low screen brightness mode (Mean = 1.802 ± SD = 0.766), medium screen brightness mode (Mean = 2.140 ± SD = 0.792), and high screen brightness mode (Mean = 2.233 ± SD = 0.881), shows that the anti-fatigue performance of the low screen brightness mode is better than the other two modes. Under the same color but different brightness situation, which includes the green group (Mean = 2.117 ± SD = 0.825), blue group (Mean = 1.892 ± SD = 0.720), red group (Mean = 2.288 ± SD = 0.860), and black group (Mean = 1.937 ± SD = 0.868), it is indicated that, in the visual anti-fatigue index dimension, red > green > black > blue, and moreover, the anti-fatigue effect of blue is the best, and the anti-fatigue effect of red is the worst.

## 4. Discussion

The research points of this paper are based on the different fatigue degrees of human vision when people are stimulated by the background brightness and different color combinations of the computer screen when they are looking at the computer screen in a dark environment without an external light source. From the data analysis, it can be concluded that EEG data reveal that the human eye will produce obvious visual fatigue when watching 12 paradigms with different brightness and colors in this environment. Eye movement data revealed that the degree of visual fatigue was lower in low brightness conditions. The Likert scale revealed that the subjects felt visual fatigue obviously in the experiment. According to the score of subjective preference, the subjects preferred blue and black to red in the low brightness mode, and preferred blue to red in the high brightness mode. Further analysis showed that in three different brightness modes of the same color, the subjects preferred the performance of green in the low brightness mode.

Previous studies have pointed out that in the dark mode, the human eye will produce a certain degree of visual fatigue when observing, working, and doing some other tasks [50]. When the human brain processes visual signals, it will produce visual fatigue [51], however, there is no significant difference between the different brightness and different colors of the paradigms when this kind of asthenopia is reflected in the EEG signal [52]. Jeong and Mathôt pointed out that the pupil of the human eye is more likely to feel visual fatigue in a brighter environment [53,54]. However, the sensitivity of the pupils to different colors is not strong, although some experiments have proved that the degree of visual fatigue of human eyes for different colors is different, for example, red is more likely to induce fatigue than green [55]. In the results, it is obvious that no matter what kind of brightness environment, the subjects always dislike red more. In other words, red can always induce stronger visual fatigue. A study shows that high stimulus seekers prefer red, whereas low stimulus seekers prefer blue [56], and high stimulus means higher visual fatigue. This also explains why in the visual anti-fatigue index of the subjects: red > green > black > blue, blue is the most popular and red is the least popular.

There are two main findings in this paper. First, the significant difference in visual fatigue is caused by different screen brightness. The difference in the visual anti-fatigue index caused by different paradigm colors is mainly reflected in the subjective data, whereas there is no significant difference from the objective data, whether eye movement data or EEG data. Second, the objective EEG data cannot reflect the characteristics of the screen brightness of the experimental environment or the color of the paradigm. Therefore, combining these two points, we find that our applications can be divided into the following categories: Firstly, a simple and objective quantitative visual fatigue index [57] is proposed, which helps to reduce the risk of eye injury and disease. Secondly, in the development of software and hardware of electronic equipment, multi-color can be combined with less traditional black and white stimulation to avoid more visual fatigue [58]. At the same time, the different screen brightness in the dark environment needs a different reaction speed to avoid reducing visual recognition performance [59]. Better products can be designed by combining hue and brightness, such as when sensing low illumination, When the brightness of the screen decreases, the background can be adjusted to blue. Thirdly, at the same time, the method proposed by Sato et al. can obtain an excellent image quality in a dark lighting environment [60], and combined with this method, it can give the viewer a better experience. Fourthly, based on the research of Zhou et al., in order to obtain the most comfortable user experience, the ambient brightness and screen brightness levels should be in the range of 13.08–62.16 lx and 20.63–75.15 CD/m^2^, respectively [61]. Combined with the data in this paper, the range of comfort experience can be expanded, because with the increase of the frequency of people using electronic devices at night [62], the increase of the use time in such a low illumination environment is inevitable. Fifthly, the SSVEP paradigm will also be used to detect visual acuity [63]. For the sake of attracting the attention of the subjects in the detection, we can combine the red with strong stimulation and the blue with a comfortable appearance in the case of appropriate brightness.

The visual fatigue detection method in this paper combines the performance analysis of two objective indicators and two subjective indicators, and proposes a representative visual anti-fatigue index, which can effectively reflect the degree of visual fatigue after the experiment to a certain extent, and has good timeliness and accuracy. For traditional detection methods, there are those which are purely focused on the subjective feelings of the data analysis method [64], and there are also objective data for the visual fatigue analysis method [65]. However, this method uses a combination of subjective and objective, such as combining the mechanism between eye movement and the focus depth adjustment function, focusing on the conflict between eye movement and the adjustment function, and the subjective feelings for visual fatigue analysis [48]. Self-report analysis combined with distance measurements [66] followed by the candidate objects are divided into principal components. The principal component analysis is used to determine the validity of each principal component. Subjective visual fatigue and multiple regression are used to predict visual fatigue [67]; the visual fatigue was quantified by the EEG and subjective method [68]. The objective factors of environmental illumination and visual angle were combined with the subjective factors of the subjects [69]. Even in the detection of asthenopia in some pathological eye diseases, the fusion method is used to measure the transmittance of the liquid crystal placed in front of the non-primary eye of the subjects and the subjective symptoms of the subjects [49]. Therefore, the combination of subjective and objective data is the mainstream method of visual fatigue detection and evaluation.

The main shortcomings of this article lie in the following three points: firstly, it is limited by the relationship between the experimental time, only three kinds of screen brightness and four kinds of paradigm colors can be matched, and the experimental design cannot be further refined, such as brightness being divided into finer elements, and color matching also being other colors, not being limited to a white background. Secondly, only eight-channel EEG acquisition equipment was used to collect the visual area data, but whether there are other nerve centers involved in the integrated impact of visual fatigue is still unclear. If some channels can be added and combined with MRI for EEG analysis, maybe we can dig out different data and get some updated findings. Thirdly, in this article, the color order in each screen brightness group is fixed, which lacks authenticity, that is to say, to ensure the performed experiment is purely authentic we must guarantee that the color order in each screen brightness group is random. Fourthly, the proposed parameter design of the visual anti-fatigue index is based on the theoretical experience of the relevant literature and the relevant practical experience in experiments. It lacks a certain mathematical derivation basis and also lacks the relevant quantitative and standardized literature references. It may be able to dig deeper into parameter optimization.

## 5. Conclusions

The results show that the paradigm color and screen brightness have a significant relationship with the visual fatigue and preferences in the low environment lighting scene which simulates the night environment. In the night environment, the low screen brightness leads to the lightest visual fatigue, and the screen brightness of the medium and high levels will cause more serious visual fatigue. As for the paradigm color, the performance of three colors (green, blue, and black), except red, in the mode of a low screen brightness are better than the other two brightness modes. Among the subjective preference scores, the participants are more likely to get lower visual fatigue in the low brightness mode, the scores of red are the lowest, which means the red color could lead to the most visual fatigue to users. In the high brightness mode, blue is more popular and red is not accepted.

This paper discusses the visual fatigue degree of human eyes with different brightness and different paradigm colors when using electronic equipment at night. The study has four application directions: Firstly, users should try to use low screen brightness to work in the case of low illumination at night, so as to avoid visual fatigue more effectively, furthermore, avoid too bright fonts when reading, such as red and green fonts. Secondly, this study can guide some electronic equipment hardware manufacturers and electronic software development companies, and make contributions to adaptive adjustment and the human–computer interaction of different colors and different screen brightness. For example, if the user’s actual working environment is dark, the screen brightness cannot be designed to be too bright in the display design. At the same time, the default font color should be as light as possible. Thirdly, this also suggests the direction of future research: experiments can be carried out on the visual fatigue of users with different screen brightness under different light environments (such as different illuminance), so as to improve the whole visual anti-fatigue system. Fourthly, the visual anti-fatigue index can be used to define and distinguish some visual anti-fatigue equipment on the market. Although it is far from being used as the industry standard, it also has a certain reference and reference significance.

There are the following points in the future research work: First, expand the scope of the paradigm color and then refine it more. Second, people not only need to use electronic equipment in the pure dark environment, but also work under lighting conditions. It is necessary to superimpose a certain light environment (such as illumination and color temperature) to study visual fatigue. Third, for EEG data analysis of visual fatigue, in addition to CCA, some popular tools such as information entropy can also be used to dig some different information.

## Figures and Tables

**Table 1:** Design of Paradigms.

Number of Paradigm	Screen Brightness	Explanation	Abbreviation
1	0%	Low brightness mode—Green light flicker	LG
2	0%	Low brightness mode—Blue light flicker	LBE
3	0%	Low brightness mode—Red light flicker	LR
4	0%	Low brightness mode—Black light flicker	LBK
5	50%	Medium brightness mode—Black light flicker	MBK
6	50%	Medium brightness mode—Blue light flicker	MBE
7	50%	Medium brightness mode—Green light flicker	MG
8	50%	Medium brightness mode—Red light flicker	MR
9	100%	High brightness mode—Blue light flicker	HBE
10	100%	High brightness mode—Black light flicker	HBK
11	100%	High brightness mode—Red light flicker	HR
12	100%	High brightness mode—Green light flicker	HG

**Figure A1:**
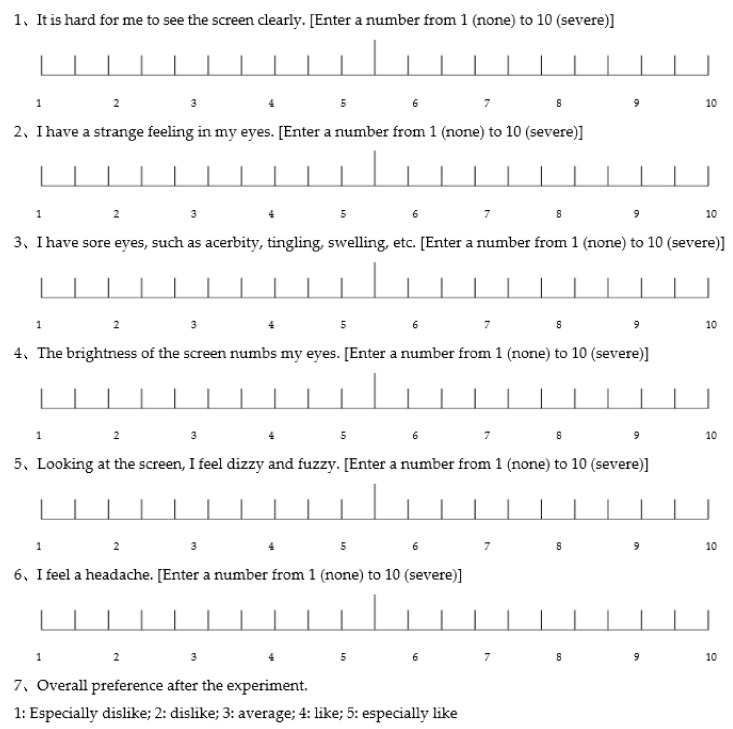
Likert Scale of Visual Fatigue Evaluation.

## Data Availability

The raw data supporting the conclusions of this article will be made available by the authors, without undue reservation, to any qualified researcher.

## Appendix A

**Table A1:** Bonferroni Post-Hoc Analysis of the First Three Trials of Pupil Diameter among Twelve Paradigms.

Paradigm (Value of P)	2	3	4	5	6	7	8	9	10	11	12
1	1.000	1.000	1.000	0.013 *	0.071	0.029 *	0.002 **	0.017 *	0.005 **	0.005 **	0.023 *
2	-	1.000	1.000	0.009 **	0.058	0.014 *	<0.001 ***	0.005 **	0.003 **	<0.001 ***	0.007 **
3	-	-	1.000	0.054	0.407	0.440	0.003 **	0.039 *	0.075	0.002 **	0.032
4	-	-	-	0.025 *	0.123	0.058	0.025 *	0.037 *	0.002 **	0.005 **	0.029 *
5	-	-	-	-	1.000	1.000	1.000	1.000	1.000	1.000	1.000
6	-	-	-	-	-	1.000	1.000	1.000	1.000	1.000	1.000
7	-	-	-	-	-	-	1.000	1.000	1.000	1.000	1.000
8	-	-	-	-	-	-	-	1.000	1.000	1.000	1.000
9	-	-	-	-	-	-	-	-	1.000	1.000	1.000
10	-	-	-	-	-	-	-	-	-	1.000	1.000
11	-	-	-	-	-	-	-	-	-	-	1.000
12	-	-	-	-	-	-	-	-	-	-	-

*** *p* < 0.001; ** *p* < 0.01; * *p* < 0.05.

**Table A2:** Bonferroni Post-Hoc Analysis of Likert Scale of Visual Fatigue Scale among Twelve Paradigms.

Paradigm (Value of P)	2	3	4	5	6	7	8	9	10	11	12
1	1.000	1.000	1.000	1.000	1.000	1.000	1.000	1.000	1.000	0.241	0.149
2	-	0.308	1.000	0.498	1.000	1.000	0.285	1.000	1.000	0.171	0.059
3	-	-	1.000	1.000	1.000	1.000	1.000	1.000	1.000	1.000	1.000
4	-	-	-	1.000	1.000	1.000	1.000	1.000	1.000	0.837	1.000
5	-	-	-	-	1.000	1.000	1.000	1.000	1.000	1.000	1.000
6	-	-	-	-	-	1.000	1.000	1.000	1.000	1.000	1.000
7	-	-	-	-	-	-	1.000	1.000	1.000	1.000	1.000
8	-	-	-	-	-	-	-	1.000	1.000	1.000	1.000
9	-	-	-	-	-	-	-	-	1.000	1.000	1.000
10	-	-	-	-	-	-	-	-	-	0.035 *	0.480
11	-	-	-	-	-	-	-	-	-	-	1.000
12	-	-	-	-	-	-	-	-	-	-	-

* *p* < 0.05.

**Table A3:** Bonferroni Post-Hoc Analysis of Likert Scale of Subjective Preference among Twelve Paradigms.

Paradigm (Value of P)	2	3	4	5	6	7	8	9	10	11	12
1	1.000	1.000	1.000	1.000	1.000	1.000	1.000	1.000	1.000	0.073	1.000
2	-	0.508	1.000	1.000	1.000	0.457	0.085	1.000	1.000	0.001 **	0.066
3	-	-	1.000	1.000	1.000	1.000	1.000	1.000	1.000	1.000	1.000
4	-	-	-	1.000	1.000	1.000	0.257	1.000	1.000	0.019 *	0.294
5	-	-	-	-	1.000	1.000	1.000	1.000	1.000	1.000	1.000
6	-	-	-	-	-	1.000	0.049 *	1.000	1.000	0.165	1.000
7	-	-	-	-	-	-	1.000	1.000	1.000	0.689	1.000
8	-	-	-	-	-	-	-	1.000	0.087	1.000	1.000
9	-	-	-	-	-	-	-	-	1.000	0.032 *	1.000
10	-	-	-	-	-	-	-	-	-	0.014 *	1.000
11	-	-	-	-	-	-	-	-	-	-	1.000
12	-	-	-	-	-	-	-	-	-	-	-

** *p* < 0.01; * *p* < 0.05.

**Table A4:** Bonferroni Post-Hoc Analysis of Pupil Diameter at Fatigue Level 1 among Twelve Paradigms.

Paradigm (Value of P)	2	3	4	5	6	7	8	9	10	11	12
1	1.000	1.000	0.263	0.336	1.000	0.004 **	0.018 *	1.000	0.044 *	0.020 *	0.134
2	-	1.000	1.000	0.010 *	0.649	0.001 **	0.001 **	1.000	0.001 **	<0.001 ***	0.006 **
3	-	-	0.510	0.211	1.000	0.011 *	0.004 **	1.000	0.048 *	0.004 **	0.037 *
4	-	-	-	0.001 **	0.154	<0.001 ***	<0.001 ***	1.000	<0.001 ***	<0.001 ***	<0.001 ***
5	-	-	-	-	1.000	0.378	1.000	1.000	1.000	1.000	1.000
6	-	-	-	-	-	1.000	1.000	1.000	1.000	1.000	1.000
7	-	-	-	-	-	-	1.000	1.000	1.000	1.000	1.000
8	-	-	-	-	-	-	-	1.000	1.000	1.000	1.000
9	-	-	-	-	-	-	-	-	1.000	1.000	1.000
10	-	-	-	-	-	-	-	-	-	1.000	1.000
11	-	-	-	-	-	-	-	-	-	-	1.000
12	-	-	-	-	-	-	-	-	-	-	-

*** *p* < 0.001; ** *p* < 0.01; * *p* < 0.05.

**Table A5:** Bonferroni Post-Hoc Analysis of Pupil Diameter at Fatigue Level 4 among Twelve Paradigms.

Paradigm (Value of P)	2	3	4	5	6	7	8	9	10	11	12
1	1.000	1.000	0.647	0.031 *	0.453	0.001 **	0.002 **	1.000	0.003 **	0.001 **	0.010 *
2	-	1.000	1.000	0.004 **	0.224	0.002 **	<0.001 ***	0.876	0.002 **	<0.001 ***	0.001 **
3	-	-	0.390	0.088	1.000	0.014 *	0.001 **	1.000	0.072	0.002 **	0.042 *
4	-	-	-	<0.001 ***	0.098	<0.001 ***	<0.001 ***	0.494	<0.001 ***	<0.001 ***	<0.001 ***
5	-	-	-	-	1.000	1.000	1.000	1.000	1.000	1.000	1.000
6	-	-	-	-	-	1.000	1.000	1.000	1.000	1.000	1.000
7	-	-	-	-	-	-	1.000	1.000	1.000	1.000	1.000
8	-	-	-	-	-	-	-	1.000	1.000	1.000	1.000
9	-	-	-	-	-	-	-	-	1.000	1.000	1.000
10	-	-	-	-	-	-	-	-	-	1.000	1.000
11	-	-	-	-	-	-	-	-	-	-	1.000
12	-	-	-	-	-	-	-	-	-	-	-

*** *p* < 0.001; ** *p* < 0.01; * *p* < 0.05.

## References

[1] Liang Y., Zhao C.Z., Yuan H., Chen Y., Zhang W., Huang J.Q., Yu D., Liu Y., Titirici M.M., Chueh Y.L.J.I. (2019). A review of rechargeable batteries for portable electronic devices. InfoMat.

[2] Ellahi A., Khalil M.S., Akram F. (2011). Computer users at risk: Health disorders associated with prolonged computer use. J. Bus. Econ. Manag..

[3] Ukai K., Howarth P.A. (2008). Visual fatigue caused by viewing stereoscopic motion images: Background, theories, and observations. Displays.

[4] Rideout V.J., Foehr U.G., Roberts D.F. (2010). Generation M^2^: Media in the Lives of 8-to 18-Year-Olds.

[5] Rosenfield M. (2011). Computer vision syndrome: A review of ocular causes and potential treatments. Ophthalmic Physiol. Opt..

[6] Papanastasiou G., Drigas A., Skianis C., Lytras M. (2020). Brain computer interface based applications for training and rehabilitation of students with neurodevelopmental disorders. A literature review. Heliyon.

[7] Baek H.J., Chang M.H., Heo J., Park K.S. (2019). Enhancing the Usability of Brain-Computer Interface Systems. Comput. Intell. Neurosci..

[8] Larion M., Munteanu F., Galatanu C.D. Adverse Light Environment: A New Challenge. Proceedings of the 2020 International Conference and Exposition on Electrical And Power Engineering (EPE).

[9] Herbig B., Schneider A., Nowak D. (2016). Does office space occupation matter? The role of the number of persons per enclosed office space, psychosocial work characteristics, and environmental satisfaction in the physical and mental health of employees. Indoor Air.

[10] Shantakumari N., Eldeeb R., Sreedharan J., Gopal K. (2014). Computer use and vision. related problems among university students in Ajman, United Arab Emirate. Ann. Med. Health Sci. Res..

[11] Schwartz G.S. (2008). Around the Eye in 365 Days.

[12] Golmohammadi R., Pirmoradi Z., Torghabeh M.M., Fardmal J. (2020). Lighting and color temperature assessment in the office workplaces and relationship to visual fatigue. Iran. Occup. Health.

[13] Yang F., Qin X.-f., Zhai L.-b. (2015). Control system and control method for automatic adjustment of outdoor LED display brightness. Ubiquitous Computing Application and Wireless Sensor.

[14] Pedersen L.A., Einarsson S.S., Rikheim F.A., Sandnes F.E. User Interfaces in Dark Mode During Daytime–Improved Productivity or Just Cool-Looking?. Proceedings of the International Conference on Human-Computer Interaction.

[15] Na N., Suk H.J. (2014). Adaptive luminance contrast for enhancing reading performance and visual comfort on smartphone displays. Opt. Eng..

[16] Vossen F.M., Aarts M.P.J., Debije M.G. (2016). Visual performance of red luminescent solar concentrating windows in an office environment. Energy Build..

[17] Chen H.W., Lee J.H., Lin B.Y., Chen S., Wu S.T. (2018). Liquid crystal display and organic light-emitting diode display: Present status and future perspectives. Light. Sci. Appl..

[18] Xing D., Ouni A., Chen S., Sahmoud H., Gordon J., Shapley R. (2015). Brightness–color interactions in human early visual cortex. J. Neurosci..

[19] Celik T. (2012). Two-dimensional histogram equalization and contrast enhancement. Pattern Recognit..

[20] Tian Q., Ran L., Zhao C., Zhou Z., Wu H. Effects of Screen Brightness on Visual Performance Under Different Environments. Proceedings of the International Conference on Applied Human Factors and Ergonomics.

[21] Silva S., Santos B.S., Madeira J. (2011). Using color in visualization: A survey. Comput. Graph.-Uk.

[22] Dong G.P., Xiao X.D., Zhang L.L., Ma Z.J., Bao X., Peng M.Y., Zhang Q.Y., Qiu J.R. (2011). Preparation and optical properties of red, green and blue afterglow electrospun nanofibers. J. Mater. Chem..

[23] Law D., Wong C., Yip J. (2012). How does visual merchandising affect consumer affective response? An intimate apparel experience. Eur. J. Mark..

[24] Rezeika A., Benda M., Stawicki P., Gembler F., Saboor A., Volosyak I. (2018). Brain–computer interface spellers: A review. Brain Sci..

[25] Nourmohammadi A., Jafari M., Zander T.O. (2018). A survey on unmanned aerial vehicle remote control using brain–computer interface. IEEE Trans. Human-Machine Syst..

[26] Liu K., Yu Y., Liu Y., Zhou Z. A Speedy Brain-Computer Interface Combined with Computer Vision. Proceedings of the International Conference on Cognitive Based Information Processing and Applications (CIPA 2021).

[27] Da Col S. (2021). Mixed Reality Application for Inspection and Validation in Industrial Environments: Human Performance and Brain-Computer Interface Advantages over Gestures. Ph.D. Thesis.

[28] (2021). Attentional load classification in multiple object tracking task using optimized support vector machine classifier: A step towards cognitive brain–computer interface. J. Med. Eng. Technol..

[29] Guo Y., Freer D., Deligianni F., Yang G.Z. (2022). Eye-Tracking for Performance Evaluation and Workload Estimation in Space Telerobotic Training. IEEE Trans. Human-Machine Syst..

[30] Kubacki A. (2021). Use of Force Feedback Device in a Hybrid Brain-Computer Interface Based on SSVEP, EOG and Eye Tracking for Sorting Items. Sensor.

[31] Song K., Zhou L., Wang H. (2021). Deep Coupling Recurrent Auto-Encoder with Multi-Modal EEG and EOG for Vigilance Estimation. Entropy.

[32] Yin E., Zhou Z., Jiang J., Yu Y., Hu D. (2014). A dynamically optimized SSVEP brain–computer interface (BCI) speller. IEEE Trans. Biomed. Eng..

[33] Yin E., Zeyl T., Saab R., Chau T., Hu D., Zhou Z. (2015). A hybrid brain–computer interface based on the fusion of P300 and SSVEP scores. IEEE Trans. Neural Syst. Rehabil. Eng..

[34] Society A.C.N. (2006). Guideline 5: Guidelines for standard electrode position nomenclature. Am. J. Electroneurodiagn. Technol..

[35] Almoqbel F.M., Yadav N.K., Leat S.J., Head L.M., Irving E.L. (2011). Effects of sweep VEP parameters on visual acuity and contrast thresholds in children and adults. Graefes Arch. Clin. Exp. Ophthalmol.

[36] Ng K.B., Bradley A.P., Cunnington R. (2012). Stimulus specificity of a steady-state visual-evoked potential-based brain–computer interface. J. Neural Eng..

[37] Chen Y.C., Yeh S.L. (2009). Catch the moment: Multisensory enhancement of rapid visual events by sound. Exp. Brain Res..

[38] Owens M., Koster E.H.W., Derakshan N. (2013). Improving attention control in dysphoria through cognitive training: Transfer effects on working memory capacity and filtering efficiency. Psychophysiology.

[39] Xie X.J., Song F.H., Liu Y., Wang S.R., Yu D. (2021). Study on the Effects of Display Color Mode and Luminance Contrast on Visual Fatigue. IEEE Access.

[40] Kim T., Lee E.C. (2020). Experimental Verification of Objective Visual Fatigue Measurement Based on Accurate Pupil Detection of Infrared Eye Image and Multi-Feature Analysis. Sensors.

[41] Xie J., Xu G., Wang J., Li M., Han C., Jia Y. (2016). Effects of Mental Load and Fatigue on Steady-State Evoked Potential Based Brain Computer Interface Tasks: A Comparison of Periodic Flickering and Motion-Reversal Based Visual Attention. PLoS ONE.

[42] Zheng X., Xu G., Zhang Y., Liang R., Zhang K., Du Y., Xie J., Zhang S. (2020). Anti-fatigue Performance in SSVEP-Based Visual Acuity Assessment: A Comparison of Six Stimulus Paradigms. Front. Hum. Neurosci..

[43] Lin Z., Zhang C., Wu W., Gao X. (2006). Frequency recognition based on canonical correlation analysis for SSVEP-based BCIs. IEEE Trans. Biomed. Eng..

[44] Kalunga E., Djouani K., Hamam Y., Chevallier S., Monacelli E. SSVEP enhancement based on Canonical Correlation Analysis to improve BCI performances. Proceedings of the 2013 Africon, Pointe aux Piments.

[45] Bin G., Gao X., Yan Z., Hong B., Gao S. (2009). An online multi-channel SSVEP-based brain–computer interface using a canonical correlation analysis method. J. Neural Eng..

[46] Nakanishi M., Wang Y., Wang Y.T., Jung T.P. (2015). A Comparison Study of Canonical Correlation Analysis Based Methods for Detecting Steady-State Visual Evoked Potentials. PLoS ONE.

[47] Cao T., Wan F., Wong C.M., da Cruz J.N., Hu Y. (2014). Objective evaluation of fatigue by EEG spectral analysis in steady-state visual evoked potential-based brain-computer interfaces. Biomed. Eng. Online.

[48] Yano S., Ide S., Mitsuhashi T., Thwaites H. (2002). A study of visual fatigue and visual comfort for 3D HDTV/HDTV images. Displays.

[49] Hirota M., Yada K., Morimoto T., Endo T., Miyoshi T., Miyagawa S., Hirohara Y., Yamaguchi T., Saika M., Fujikado T. (2020). Objective evaluation of visual fatigue in patients with intermittent exotropia. PLoS ONE.

[50] Lambooij M., Ijsselsteijn W., Fortuin M., Heynderickx I. (2009). Visual Discomfort and Visual Fatigue of Stereoscopic Displays: A Review. J. Imaging Sci. Technol..

[51] Cai T., Zhu H., Xu J., Wu S., Li X., He S. (2017). Human cortical neural correlates of visual fatigue during binocular depth perception: An fNIRS study. PLoS ONE.

[52] Zhang N., Zhou Z., Liu Y., Yin E., Jiang J., Hu D. (2019). A Novel Single-Character Visual BCI Paradigm With Multiple Active Cognitive Tasks. IEEE Trans. Biomed. Eng..

[53] Jeong H. (2012). A comparison of the influence of electronic books and paper books on reading comprehension, eye fatigue, and perception. Electron. Libr..

[54] Mathôt S. (2018). Pupillometry: Psychology, physiology, and function. J. Cogn..

[55] Osaka N. (1985). The Effect of Vdu Color on Visual Fatigue in the Fovea and Periphery of the Visual-Field. Displays.

[56] Nelson J.G., Pelech M.T., Foster S.F. (1984). Color Preference and Stimulation Seeking. Percept. Mot. Ski..

[57] Lee C.-C., Chiang H.-S., Hsiao M.-H. (2021). Effects of screen size and visual presentation on visual fatigue based on regional brain wave activity. J. Supercomput..

[58] Sato Y., Kitamura Y., Hirata T., Bao Y. (2021). Investigation of Visual Stimulus Signals Using Hue Change for SSVEP. Appl Sci-Basel.

[59] Yao J., Hu Z.L., Jiang H.Y., Zhao X.C. (2021). Visual recognition efficiency of handheld infrared thermometer interface information under low ambient illuminance. Int. J. Ind. Ergon..

[60] Na S.H., Min W.K., Kim D.H., Hwang H.W., Ha Y.M., Kim H.J. (2021). Enhancement of picture quality on ultra-low brightness by optimizing the electrical potential required for OLED charging in the AMOLED displays. J. Inform. Disp..

[61] Zhou Y., Shi H.Y., Chen Q.W., Ru T.T., Zhou G.F. (2021). Investigation of the Optimum Display Luminance of an LCD Screen under Different Ambient Illuminances in the Evening. Appl. Sci..

[62] García-Santillán A., Espinosa-Ramos E. (2021). Addiction to the Smartphone in High School Students: How It’s in Daily Life?. Contemp. Educ. Technol..

[63] Zheng X., Xu G., Du C., Yan W., Tian P., Zhang K., Liang R., Han C., Zhang S. (2021). Real-time, precise, rapid and objective visual acuity assessment by self-adaptive step SSVEPs. J. Neural Eng..

[64] Li H.-C.O., Seo J., Kham K., Lee S. (2008). Method of measuring subjective 3D visual fatigue: A five-factor model. Digital Holography and Three-Dimensional Imaging.

[65] Song M.C., Li L., Guo J.T., Liu T., Li S.Y., Wang Y.T., ul Ain Q., Wang J. (2020). A new method for muscular visual fatigue detection using electrooculogram. Biomed. Signal Process..

[66] Lambooij M., Fortuin M., Ijsselsteijn W., Evans B., Heynderickx I. (2010). Measuring visual fatigue and visual discomfort associated with 3-D displays. J. Soc. Inf. Disp..

[67] Choi J., Kim D., Choi S., Sohn K. (2012). Visual fatigue modeling and analysis for stereoscopic video. Opt. Eng..

[68] Kim Y.-J., Lee E.C. EEG based comparative measurement of visual fatigue caused by 2D and 3D displays. Proceedings of the International Conference on Human-Computer Interaction.

[69] Wu H.-C. (2012). Visual fatigue and performances for the 40-min mixed visual work with a projected screen. Ergon. Open J..

